# Patient Satisfaction in Doctor Patient Communication in a Tertiary Care Hospital of Kathmandu: A Descriptive Cross-Sectional Study

**DOI:** 10.31729/jnma.6289

**Published:** 2021-04-30

**Authors:** Milan Chandra Khanal, Lochan Karki, Badri Rijal, Pramod Joshi, Navindra Raj Bista, Bikash Nepal, Krishna Rana, Prabesh Lamichhane

**Affiliations:** 1 Shepherd College, New Baneshwor, Kathmandu; 2 Department of Medicine, National Academy of Medical Sciences, Kathmandu, Nepal; 3 Department of Orthopaedics, All Nepal Hospital Private Limited, Samakhushi, Kathmandu; 4 Department of Orthopaedics, National Trauma Hospital, Kathmandu, Nepal; 5 Department of Anaesthesia, Tribhuwan University-Teaching Hospital, Kathmandu, Nepal; 6 Department of Surgery, National Academy of Medical Sciences, Kathmandu, Nepal; 7 All Nepal Hospital Private Limited, Samakhushi, Nepal; 8 Nepal Medical Association, Bagbazar, Kathmandu, Nepal

**Keywords:** *communication*, *patient*, *physicians*, *satisfaction*, *treatment*

## Abstract

**Introduction:**

Communication is an important aspect of the medical profession. Doctor-patient communication plays a significant role in health care delivery. This study aims to find outpatient department patient satisfaction in doctor-patient communication in a tertiary care hospital in Kathmandu, Nepal.

**Methods:**

A descriptive cross-sectional study was conducted in the outpatient department of a tertiary care hospital in Kathmandu, Nepal in the month of August 2019. Validated questionnaire of Patient Satisfaction Questionnaire, consisting of 80 items, originally developed by Willis H. Ware and his colleagues were used and distributed to the patient in the outpatient department of the hospital. Their satisfaction level for doctor-patient communication was assessed on a five-point scale. The questionnaire was distributed randomly to the patient attending the hospital outpatient department during one month period.

**Results:**

Out of the total participants, 420 (96%) at 95% CI (95.07-96.93) respondents reported that they were satisfied regarding communication with their doctors. Among the patients, 109 (24.0%) visited the department of medicine followed by obstetrics and gynaecology 85 (19.4%).

**Conclusions:**

The majority of participants were found to be satisfied with the doctor-patient communication. While this study has shown that the communication in the doctor-patient relationship was seen to be satisfactory, this might not show the generalized picture of the country. We should also think of ways to further improve the communication in our hospitals.

## INTRODUCTION

Doctor-patient communication plays a significant role in health care delivery. It affects the patient's recovery, quality of health care delivery, and ultimately the doctor's success. This communication is different as it is often non-voluntarily and involves parties that are unequal in position. Excellent doctor-patient communication ensures that the patient and the patient party understands the condition of the patient accurately so that they can make appropriate decisions according to the weight of the condition.^1^

Because physicians' communication and perceptions are related to outcomes, it is critically important to account for variability in physicians' behaviour as well as understand why different doctors talk and perceive different patients differently. With such an understanding, researchers and educators will be better positioned to effectively examine relationships between communication and outcomes as well as design interventions for improving the quality of health care.^2^ Good doctor-patient concordance leads to better trust in the physician, which in turn leads to better patient enablement, irrespective of the socio-cultural determinants.^3^

This study aims to find outpatient department (OPD) patient satisfaction in doctor-patient communication in a tertiary care hospital of Kathmandu, Nepal.

## METHODS

This is a descriptive cross-sectional study that was conducted in Civil Service Hospital, Minbhawan in the month of August 2019. The ethical approval was taken from Nepal Health Research Council (NHRC) (Reference Number: 2934). The study was conducted in the OPD of the hospital. Verbal and written consent was taken from each patient. A closed seal envelope was given for the selected patient. Validated questionnaire of Patient Satisfaction Questionnaire, consisting of 80 items, originally developed by Willis H. Ware and his colleagues4 were used to formulate the questionnaire and were distributed to the patient in the outpatient department of the hospital. The study followed the sample size calculation, sampling, collection of samples, scrutinizing of samples with giving a score based on the type of response, collection, and analysis of the data. All the patients coming to the outpatient department of the civil hospital were included in the study. A convenient sampling technique was used. Any patient who didn't want to participate in the study was excluded. They were explained about the questionnaire and provided a separate space with the questionnaire to fill-up the form. Around five minutes were allocated for filling each questionnaire. They were de-identified but the hospital number was recorded in the forms.

The sample size of this study is calculated using the formula,

$$\begin{array}{ll} \text{n} & ={\text{Z}}^{\text{2}}\times \text{p}\times \text{q}/{\text{e}}^{\text{2}}\\ & ={\left(1.96\right)}^{2}\times 0.5\times (1-0.5)/{\left(0.05\right)}^{\text{2}}\\ & =385 \end{array}$$

where,

Z = 1.96 at 95% Confidence Interval
p = assuming 50% patient are satisfied with communication with doctor, 0.5
q = (1-p)
e = 5% margin of error

Therefore, the calculated sample size was 384. After accounting for 10% non-response rate, the sample size was determined to 424. However, the questionnaire was distributed to 450 patients at the hospital to increase the precision.

The forms were collected after they were filled, and then was assembled, and analyzed. Data were collected and analyzed in Microsoft Excel 2019 in Windows 10. The descriptive statistical analysis was done. Mean, median and standard deviation were calculated for continuous data whereas frequency and proportions were calculated for binary data.

## RESULTS

A patient satisfaction questionnaire was distributed to 450 patients. A total of 437 patient returned a fully completed questionnaire. Among them, almost all 420 (96%) at 95% CI (95.07 - 96.93) stated that they were satisfied with the communication with the doctor. The age of the patients in the study was from below 15 to above 60 years. The majority 132 (31.3%) of respondents were at the age of 31 to 45 years, followed by the age of 16-30 years; 117 (26.7%), 45-59 years; 105 (24.0%), above 60 years 60 (13.7%), and less than 15 years were 23 (5.2%). Total 267 (61%) were female and 167 (39%) were male in the study.

**Figure 1.**
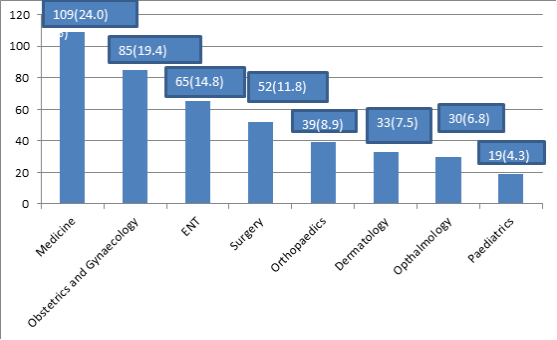
Departments visited by patients n (%).

Among the patients, 109 (24.0%) visited the department of medicine followed by obstetrics and gynaecology 85 (19.4%) (Figure 1). The majority of respondents spoke Nepali 361 (83%), followed by Newari 28 (6%), Maithili 25 (6%) other languages 22 (5%) as their native-speaking language. Majority of respondents were housewife 119 (27.2%), followed by others 85 (19.4%), students 63 (14.4%), government service 52 (11.9%), business 51 (11.6%), farmers 34 (7.7%), teachers 19 (3.2%) and technical 14 (2.9%).

**Figure 2.**
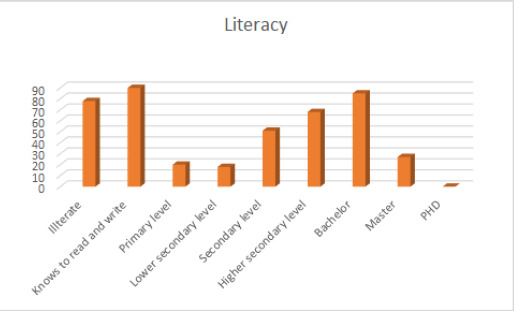
Education level of patients visiting hospital.

The majority of respondents had visited Civil Service Hospital for the first time 191 (44%), followed by more than the second time 157 (36%) and the second time 88 (20%). Most respondents were known to read and write 90 (20.6%), followed by bachelor 85 (19.4%) (Figure 2). Majority of respondents were from province number 3, 214 (48.9%), followed by province number 5, 59 (13.5%), province number 2, 47 (10.7%), province number 1, 45 (10.2%), province number 4, 33 (7.5%), province number 7, 23 (5.2%) and province number 6, 16 (3.6%).

In response to several questions given in the Patient Satisfaction Questionnaire, more than half of the participants agreed to the different questions as given below (Table 1).

**Table 1:** Response to questions as per the questionnaire.

	Agree	Strongly Agree	Not Sure	Disagree	Strongly Disagree
Did The Doctor Take Your Consent Before Sending Lab Investigations Or Other Necessary Investigations?	258 (59)	138 (32)	23 (5)	15 (3)	3 (1)
Did The Doctor Properly Explain The Medications You Are Supposed To Have?	266 (60)	142 (32)	19 (4)	17 (4)	2 (0)
Did The Doctor Make Sure That You Understood His Explanations And Instructions?	270 (62)	136 (31)	17 (4)	12 (3)	2 (0)
In Your Opinion, Does The Doctor Have A Reassuring Attitude And Way Of Talking?	233 (53)	195 (45)	5 (1)	4 (1)	0 (0)
Did The Doctor Allow You Encourage You To Express/Talk?	260 (85)	136 (13)	23 (1)	16 (1)	2 (0)
Do You Feel That The Doctor Understood You?	256 (59)	167 (38)	9 (2)	3 (0.5)	2 (0.5)
Did The Doctor Explain The Advantages And Disadvantages Of The Treatment Or Care Strategy?	264 (60)	133 (30)	22 (5)	12 (3)	6 (1)
Did The Doctor Allow You To Talk Without Interrupting You?	266 (59)	156 (35)	4 (1)	8 (2)	15 (3)
Did The Doctor Listen To You Carefully During The Consultation?	228 (52)	197 (45)	5 (1)	5 (1)	2 (1)

## DISCUSSION

The present study has explored the view of the patient regarding their communication with the doctors in the form of a questionnaire. This study was carried out through a mixed-method research approach. Information is collected through valid questionnaires with questions on demography, open, and closed-ended questions related to patient satisfaction and quality of doctor-patient communication. Likert's scoring was used to assess the degree of satisfaction. According to our study, 420 (96%) patients were satisfied with the doctors, and 17 (4%) were not satisfied with the doctors that were similar to the study done in Tribhuwan University Teaching Hospital, Maharajgung.^5^ The results from the questionnaire survey for patients show that the majority of the respondents have a positive reaction to the way doctors communicate with them. They are satisfied with the care, the consultation, the allocated time by the doctors, and the attention they received. However, it is important to note that the majority of the patients had no education. So, satisfaction in this study cannot be compared to the satisfaction level of doctor-patient communication in countries such as the UK or the USA because the literacy status on health awareness is much lower in Nepali patients. In the study conducted in Patan Academy of Health Sciences by Rajak, et al. in 2018, out of 101 patients, those who had very high (27.7%) and high level (24.8%) of satisfaction are pooled across all the categories, it shows that 52.5% were overall satisfied while remaining 47.5 % had medium to low level of satisfaction.^6^ The questionnaires also have similarities and but the result is somehow different. This difference can be due to a lack of a good sampling method in our settings while the study conducted in PAHS has a suitable sampling method, i.e., stratified random sampling, and also, the hospital setting is different. According to the study done by Smith et al., it was found that the results'' of correlation analysis indicate that higher patient satisfaction was associated with greater interview length, increases in the proportional time spent by the physician in presenting information and discussing prevention, and shorter chart review times.^7^ The increased patient understanding was associated with increases in the proportional time spent presenting both information and opinions, close physical proximity, and reduced chart review time. In the study (Physician cultural competence and patient ratings of the patient-physician relationship) done by Paez, et al. it was found that Attitudinal and behavioural components of cultural competence are important to developing higher quality, participative relationships between patients and their physicians.^8^ We did not consider cultural competence in our research, which seems to be an important factor. In our study, the questionnaires were not set for the physicians. The doctor-patient relationship should always be viewed on a two-way basis where we could not perform our best. In a similar study done by Banerjee & Sanyal, 3 patients from urban areas, who also tended to have more numbers of schooling than their rural counterparts, appeared more at ease in the intimidating surroundings of a large teaching hospital.^3^ Patients from rural backgrounds found themselves indifferent in the environment of a teaching hospital. They mainly found referrals to different departments for investigations or consultations very confusing. This was obvious from the discussion that they had among themselves and their queries to the hospital staff. It was also observed during the doctor-patient consultations that people from the lower socioeconomic status and those from rural backgrounds were more passive compared to those from urban areas, with a better education. This is also only analyzed from the perspective of patients only. Studies have found that age shapes how doctors communicate with patients, how they listen to patients and the degree to which they believe and interpret what patients say to them.^8^ Patients with a higher educational level have more skills and confidence in talking to their doctors and tend to provide more information, ask more questions and speak longer than other patients.^9^ Educated patients to seem to be more expressive and opinionated and receive more diagnostic and health information than less educated people. In the question, does the doctor have a reassuring attitude and way of talking? The majority of respondents agree 233 (53%), followed by strongly agree 195 (45%), not sure 5 (1%), disagree 4(1%) and strongly disagree 0 (0%) and in the question, Did the doctor make sure that you understood his explanations and instructions?, the majority of respondents agree 264 (60%), followed by strongly agree 133 (31%), not sure 22 (5%), disagree 12 (3%) and strongly disagree 6 (1%). These two questions the Ethos part of Rhetorical analysis where the result seems to be positive but on a similar study conducted by Duwadi EP, et al. in Nepal, the ethos aspect of the Rhetorical analysis has no satisfactory result.^10^ Our study has 267 (61%) female and 167 (39%) male, which is similar to the study done by Banerjee A, et al. According to the study done by Banerjee A, et al. out of the 198 participants in the study, 110 (55.6%) were females, and 88 (44.4%) were males.^11^ According to our study, known to read and write 90 (20.59%), followed by bachelor 85 (19.45%), illiterate 78 (17.84%), Higher Secondary level 68 (15.56%) Secondary level 51 (7.09%), Master 27 (6.17%), primary 20 (4.5%), lower secondary 18 (4.11%) and none PhD degree respondents. In a similar study done by Banerjee and Sanyal, 2012,^3^ one hundred and seven (54%) did not have an education beyond the school level, 71 (35.9%) had completed graduation, and 20 (10.1%) were postgraduates. Comparing these data, the respondents coming to our hospital setting were less qualified in their academic background. Low literacy level and health awareness of the patients consequently lead to patients being passive during medical consultation.

Since this is a single institutional study with convenient sampling, the results cannot be generalized. This increases the chances of selection bias. Also, the sample does not include patients from all the outpatient departments; there is a bias of selection of the study site. Other limitations of our study include social desirability bias and information bias. Kuppuswamy socioeconomic determinants were used in another study Banerjee, et al. 2012, we did not use this method, so we are unable to analyze the socioeconomic status scientifically.

## CONCLUSIONS

The majority of participants were found to be satisfied with the doctor-patient communication. Although the results of this study show a good outcome between the communication in the health-care setting of that hospital. Still, there can be various places where both the doctors and the hospital administration can work to further improve communication and improve healthcare delivery.
